# Colorectal anastomotic safety assessment using ICG fluorescence and flexible endoscopy (COLOSSEUM): a global survey of 1367 surgeons

**DOI:** 10.1007/s00464-026-12974-4

**Published:** 2026-07-13

**Authors:** A. Belvedere, E. Licardie, D. Sochorova, L. Boni, M. Chand, S. Perretta, S. Wexner, T. H. A. Arulampalam, S. Morales-Conde, S. F. Hardon

**Affiliations:** 1 Department of General and Emergency Surgery, IRCCS Azienda Ospedaliero-Universitaria Di Bologna, Bologna, Italy; 2 Department of General and Digestive Surgery, University Hospital Virgen Macarena, University of Sevilla, Seville, Spain; 3 Department of Surgery, Tomas Bata Hospital, Zlin, Czech Republic; 4 Department of HPB and Transplant Surgery, Freeman Hospital, Newcastle Upon Tyne, UK; 5 Department of General & Minimally Invasive Surgery, Fondazione IRCCS – Ca’ Granda - Ospedale Maggiore Policlinico di Milan, Milan, Italy; 6 Department of Advanced Minimally-Invasive Surgery, University College London Hospital, London, UK; 7 Department of Surgery, University Hospital, Strasbourg, France; 8 Department of Colorectal Surgery, Ellen Leifer Shulman and Steven Shulman Digestive Disease Center, Cleveland Clinic Florida, Weston, FL USA; 9 Department of Colorectal Surgery, Medstar Georgetown University Health, Washington, DC USA; 10 School of Medicine, Anglia Ruskin University, Chelmsford, UK; 11 Department of Surgery, Amsterdam UMC, De Boelelaan 1117, 1081HV Amsterdam, The Netherlands

**Keywords:** Colorectal anastomosis, ICG fluorescence, Endoscopy, Colorectal surgery, Anastomotic leak

## Abstract

**Aim:**

Anastomotic leakage remains a major complication in colorectal surgery. Indocyanine green (ICG) fluorescence and flexible endoscopy (FE) are used to assess perfusion and anastomotic integrity. We aimed to evaluate global practice patterns and intersurgeon variability to inform structured recommendations.

**Methods:**

This international survey was conducted between April and November 2024 within the EAES Rising Stars Academy 2023–2024. In a Delphi-like process, 53 questions addressing surgeon characteristics, institutional practice, and the use of ICG and FE were developed and distributed worldwide.

**Results:**

A total of 1367 respondents from 80 countries participated (mean age 64 ± 10 years). ICG was available at 76% of institutions, and 67% of surgeons reported using it. Timing varied: before transection 40%, after transection 7%, before anastomosis 17%, after anastomosis 25%, and both before and after 11%. A fixed dose was used by 71% and weight-based dosing by 29%. FE was performed by 51% of surgeons, with 23% reporting routine intraoperative use. Key barriers included gastroenterologist-led practice (28%), reliance on alternative techniques (20%), and lack of training (19%). Substantial variability was observed across all domains.

**Conclusions:**

This survey revealed significant heterogeneity in the use of ICG and FE in colorectal surgery. Although ICG is more widely implemented, both techniques lack standardized protocols. Moreover, FE adoption remains limited by training and access. Training should be enhanced and could represent a key area of focus for future EAES programs.

**Supplementary Information:**

The online version contains supplementary material available at https://doi.org/10.1007/s00464-026-12974-4.

Colorectal surgery is among the most frequently performed surgical subspecialties worldwide, with substantial caseloads in both elective and emergency settings. Preventing complications are critical to limit postoperative morbidity and mortality, especially in frail emergency patients and in those needing adjuvant systemic therapy, which can be delayed or withheld if major complications occur.

One of the most severe postoperative complications is anastomotic leak (AL), which substantially worsens both short-term and long-term outcomes [1]. The reported incidence of AL varies widely from 2.8 to 30 percent, with approximately 75 percent occurring in rectal anastomoses. These leaks are associated with a mortality rate of 2 to 16.4 percent and a morbidity rate of 20 to 35 percent [2]. Despite advances in surgical techniques, stapling devices, and improved perioperative optimization, AL rates have remained largely unchanged over recent decades [3].

Adequate bowel perfusion is a cornerstone for anastomotic healing, yet intraoperative assessment methods range from subjective clinical judgment to more advanced imaging technologies [4]. Indocyanine green (ICG) fluorescence imaging has gained widespread adoption over the past decade and is now the most commonly used intraoperative technique for evaluating tissue perfusion, ureteral anatomy, sentinel lymph nodes, and lymphatic pathways [5, 6]. Other innovative modalities such as laser speckle contrast imaging, hyperspectral imaging, and near-infrared spectroscopy show promise but are not implemented routinely in clinical practice [7–9].

Intraoperative flexible endoscopy (FE) offers complementary intraluminal information, including anastomotic integrity, bleeding, or venous congestion, that cannot be captured by perfusion assessment alone [10–12]. Moreover, systematic reviews and meta-analyses suggest that intraoperative FE may improve detection of anastomotic defects and reduce postoperative anastomotic complications, further supporting its role in colorectal surgery [10, 13]. However, considerable variation exists in how ICG and FE are applied in clinical practice, including differences in dose, timing of administration, imaging protocols, and indications. This lack of standardization remains an important limitation for both clinical implementation and comparison across studies [14, 15].

Increasingly, multimodal approaches combining perfusion assessment, leak testing, and intraoperative endoscopy have been proposed to optimize anastomotic safety, highlighting the complementary roles of ICG fluorescence and FE rather than viewing these techniques as isolated modalities [16]. Despite increasing evidence for anastomotic assessment, substantial variability persists regarding their availability, indications, timing, interpretation, and integration into surgical decision-making. This study aims to provide an overview of current practice patterns and variability in the use of indocyanine green (ICG) fluorescence and intraoperative flexible endoscopy (FE) for assessing bowel perfusion and anastomotic integrity.

## Methods

We conducted an international, cross-sectional snapshot survey. This project was initiated within the European Association for Endoscopic Surgery (EAES) Rising Star Academy 2023–2024.

In order to improve anastomotic health in colorectal surgery, two major topics were addressed in this survey: ICG and FE. Besides general and demographic information about the surgeons and their institutions, this survey focused on the use of ICG and FE for the assessment of anastomotic integrity. The questions focused on the level of experience with colorectal surgery (both open and minimally invasive), and the experience with ICG and FE.

The total number of questions was 53. The general part included 16 questions such as surgeons’ age, country of practice, the type of hospital, type of training, specialization, and case load. The ICG component consisted of 19 questions. These questions were grouped into four domains: availability, confidence in use, protocols in the operating room, and opinions on use. The endoscopy section consists of 18 questions. These questions were grouped into three domains: availability of FE, use in the operating room, and opinions on its use. The survey included some questions with limited responses due to the survey’s branching logic. Depending on a preceding yes or no response, certain follow-up questions were either skipped or only shown to a small subgroup of respondents; these subgroup-specific items were therefore excluded from analysis.

The questionnaire was distributed primarily via the EAES secretary, followed by distribution via other surgical professional societies like SAGES, ESCP, ISFGS, as well as social media. The survey was created using the REDCap platform (Supplemental Files).

## Results

**Fig. 1:**
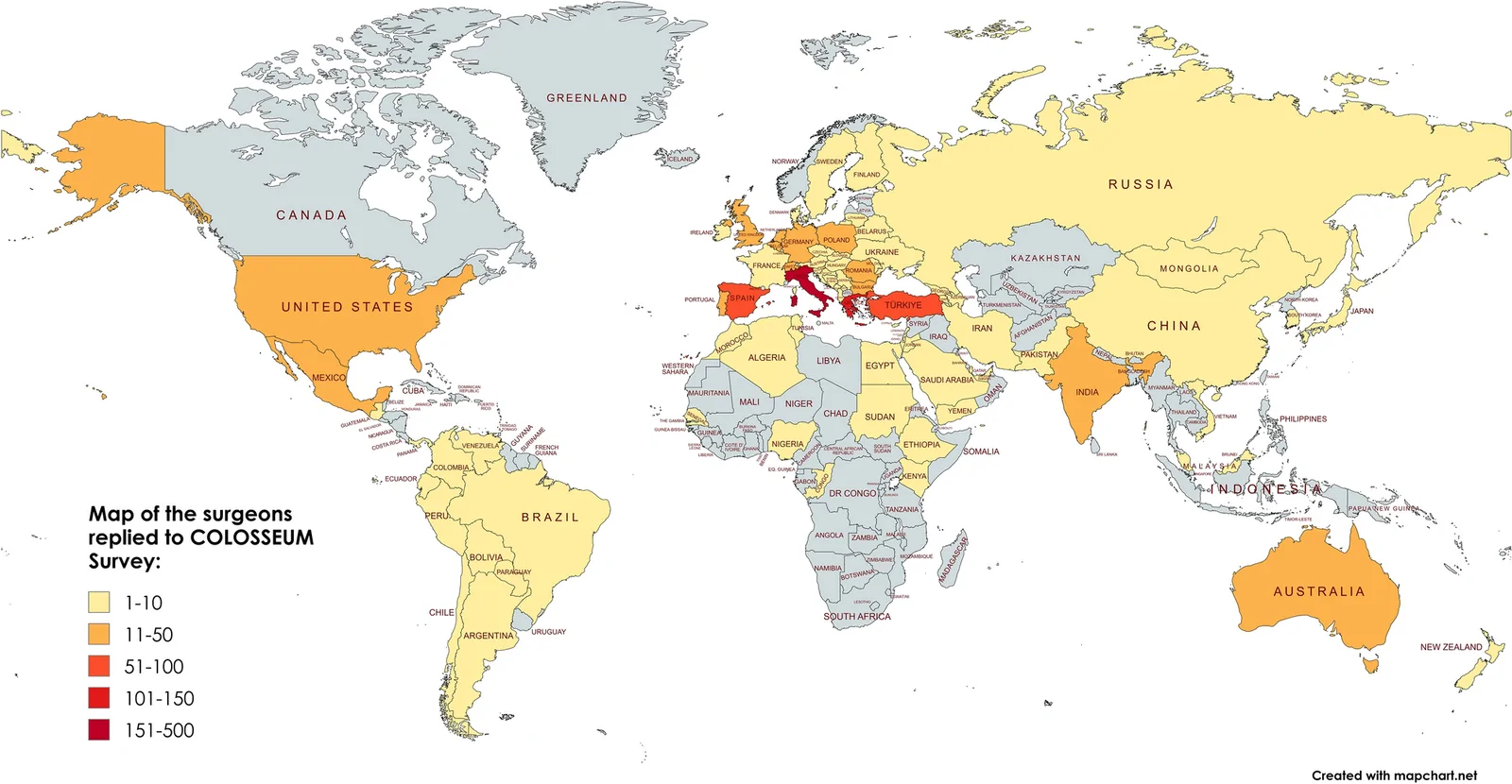
Map of the states where surgeons who responded to the survey work

A total of 1367 surgeons from 83 countries responded to this survey (Fig. 1). The mean age was 64.4 (SD 9.93) years. Respondents were predominantly affiliated with university hospitals 662 (49%), followed by public hospitals 505 (37%), and private hospitals 186 (14%). The majority had completed general surgery training 1119 (86%) and had also undertaken a colorectal surgery fellowship 745 (57%). Regarding surgical specialization, the majority identified as general 972 (39%) or colorectal 780 (31%) surgeons, while the remaining represented subspecialties such as upper gastrointestinal 207 (8%), hepatopancreatobiliary 155 (6%), bariatric 99 (4%), or abdominal wall 226 (9%) surgery (Table 1).

**Table 1:** General questions

Questions	Answers	%	%
Type of hospital	Private	186	14
	Public	505	37
	University	662	49
Have you finished general surgery training?	Yes	1119	86
	No	181	14
Have you also trained in colorectal surgery fellowship?	Yes	745	57
	No	559	43
Main specialty	General Surgeon	972	39
	Colorectal surgeon	780	31
	Upper GI	207	8
	HPB	155	6
	Bariatric surgeon	99	4
	Abdominal wall surgeon	226	9
	Other	76	3
How many elective MINIMALLY invasive colorectal resections (both for benign and oncological indications) are performed at your institution per year?	1–20	156	12
	21–50	201	15
	51–75	230	17
	76–100	192	15
	More than 100	543	41
How many elective OPEN colorectal resections (both for benign and oncological indications) are performed at your institution per year?	1–20	431	33
	21–50	372	28
	51–75	186	14
	76–100	130	10
	More than 100	205	15
What percentage of your elective colorectal resections (both for benign and oncological indications) are done as open cases?	Less than 10%	579	44
	11–25%	316	24
	26–50%	179	14
	51–75%	122	9
	76–89%	61	5
	More than 90%	52	4
What percentage of your elective colorectal resections (both for benign and oncological indications) are done as laparoscopic cases?	Less than 10%	120	9
	11–25%	121	9
	26–50%	195	15
	51–75%	254	20
	76–89%	284	22
	More than 90%	327	25
What percentage of your elective colorectal resections (both for benign and oncological indications) are done as robotic cases?	None	641	49.5
	Less than 10%	237	18
	11–25%	198	15
	26–50%	110	9
	51–75%	44	3.5
	76–89%	43	3
	More than 90%	28	2
How many anastomotic leaks after elective RIGHT colon resection occur per year in your surgical unit?	Less than 5%	1002	77
	5–10%	248	19
	11–15%	34	3
	16–20%	11	0.8
	More than 20%	2	0.2
How many anastomotic leaks after elective LEFT colon resection occur per year in your surgical unit?	Less than 5%	776	59.7
	5–10%	433	33.3
	11–15%	75	5.8
	16–20%	15	1
	More than 20%	2	0.2
How many anastomotic leaks after elective RECTAL surgery occur per year in your surgical unit?	Less than 5%	443	34
	5–10%	603	46.4
	11–15%	194	14.9
	16–20%	50	3.9
	More than 20%	10	0.8
How do you currently check the perfusion of the colon BEFORE the anastomosis? (multiple choice)	Visual inspection of the colon “color”	1016	30.6
	ICG	762	23
	Hyperspectral imaging	21	0.6
	Visually checking the pulse of the artery branch in the mesentery	460	13.9
	Palpatory checking the pulse of the artery branch in the mesentery (in open cases)	453	13.6
	Check if the colon blind ends bleed after making a small cut	450	13.6
	Endoscopic inspection	143	4.3
	Other	13	0.4
How do you currently check the perfusion of the colon of the colon “color” AFTER the anastomosis?	Visual inspection	867	44
	ICG	592	30
	Hyperspectral imaging	20	1
	Intraoperative endoscopy	279	14
	Don’t check anymore	181	9
	other	37	2

**Fig. 2:**
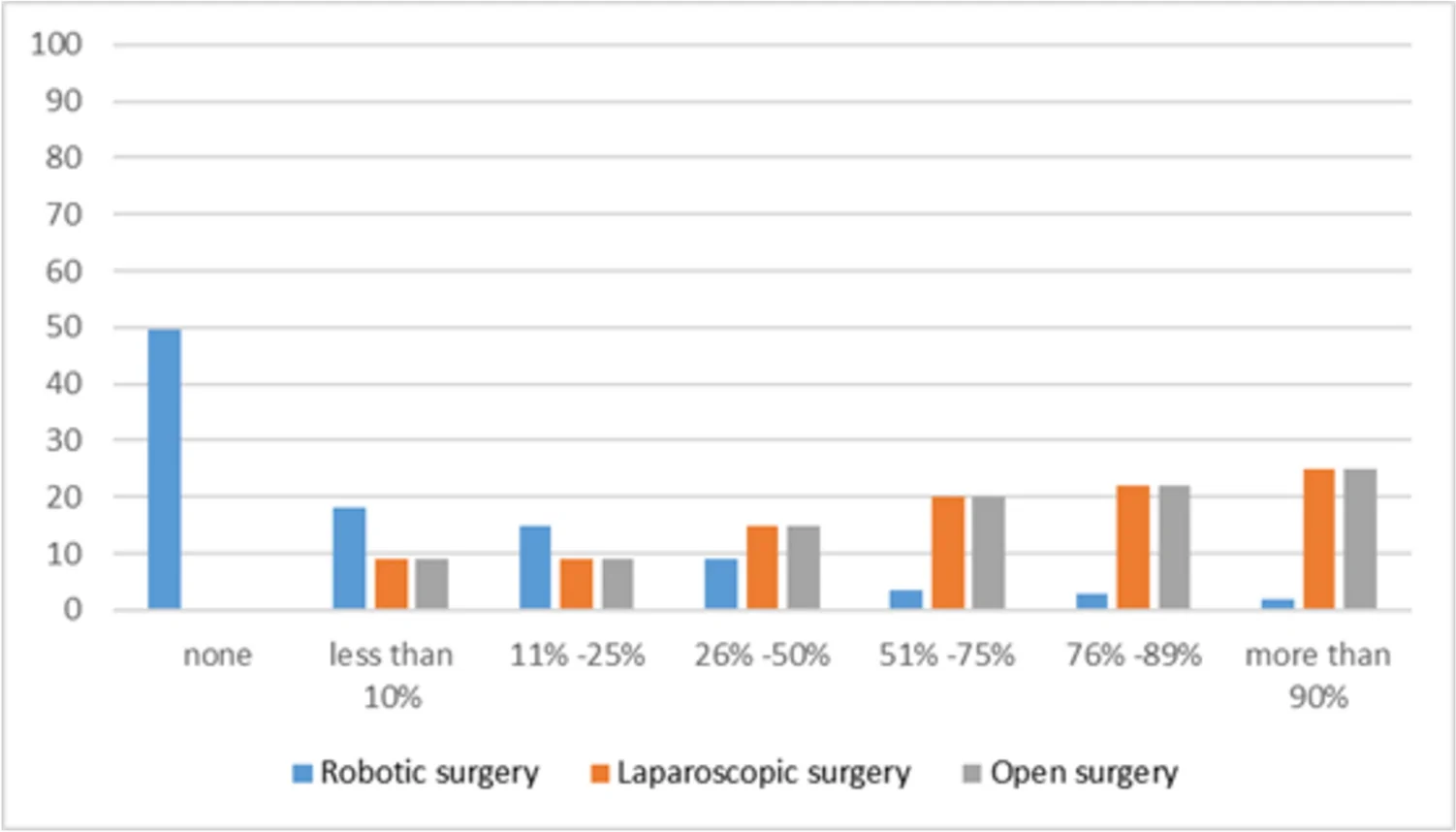
Percentage of elective colorectal resections done as open, laparoscopic and robotic cases per year in the hospital where 1367 surgeons of the survey work

Institutional case volumes for elective minimally invasive colorectal resections showed that 543 (41%) of respondents performed more than 100 cases annually. In contrast, open resections were less frequent: only 205 (15%) reported > 100 open resections per year, while a third performed fewer than 20. A significant shift toward laparoscopy was evident: 862 (67%) respondents reported ≥ 51% of resections were laparoscopic, and 327 (25%) indicated that > 90% were performed laparoscopically. Robotic surgery, however, showed more variation: 641 (50%) reported not performing any robotic resections, while only 28 (2%) reached > 90% adoption of robotic surgery (Fig. 2).

**Fig. 3:**
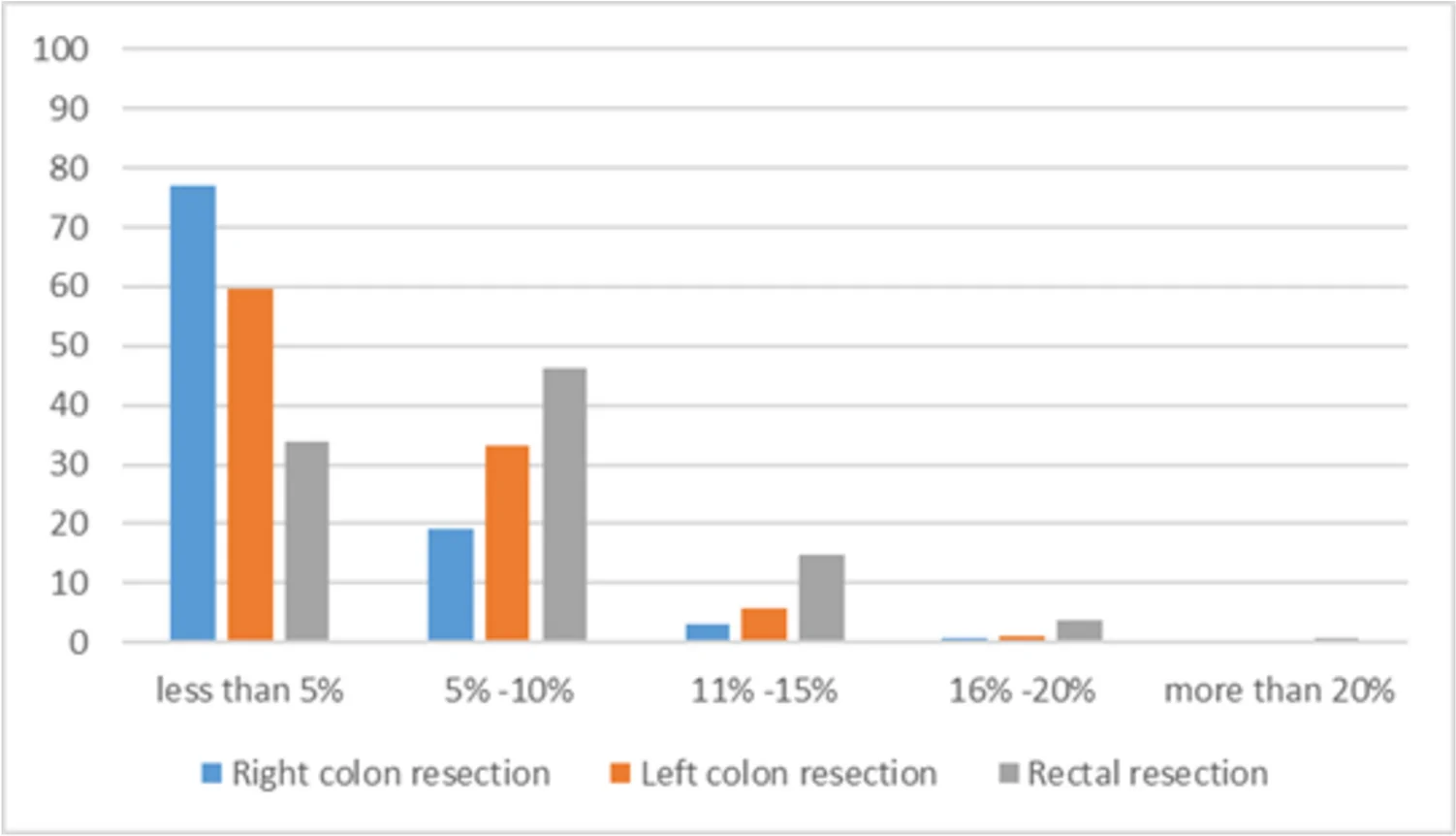
The percentage of anastomotic leak after colon or rectum resection in elective cancer surgery per year in the hospital where 1367 surgeons of the survey work

Reported rates of anastomotic leakage were generally low for right and left colectomies, with 1002 (77%) and 776 (60%) respectively stating < 5% leak rates. In rectal surgery, however, the leakage rate was notably higher: only 443 (34%) reported a < 5% leak rate, while 603 (46%) reported 5–10%, and 254 (20%) reported > 10% (Fig. 3).

Perfusion assessment strategies varied. Before anastomosis, visual inspection 1016 (30.6%), ICG fluorescence 762 (23%), and arterial pulse checking 913 (27,5%) were most commonly used. Post-anastomosis, visual inspection remained dominant for 867 (44%), while ICG was used by 592 (30%), and intraoperative endoscopy by 279 (14%). Use of advanced techniques like hyperspectral imaging was rare 20 (≤ 1%).

### Current use of ICG to assess anastomotic integrity

**Fig. 4:**
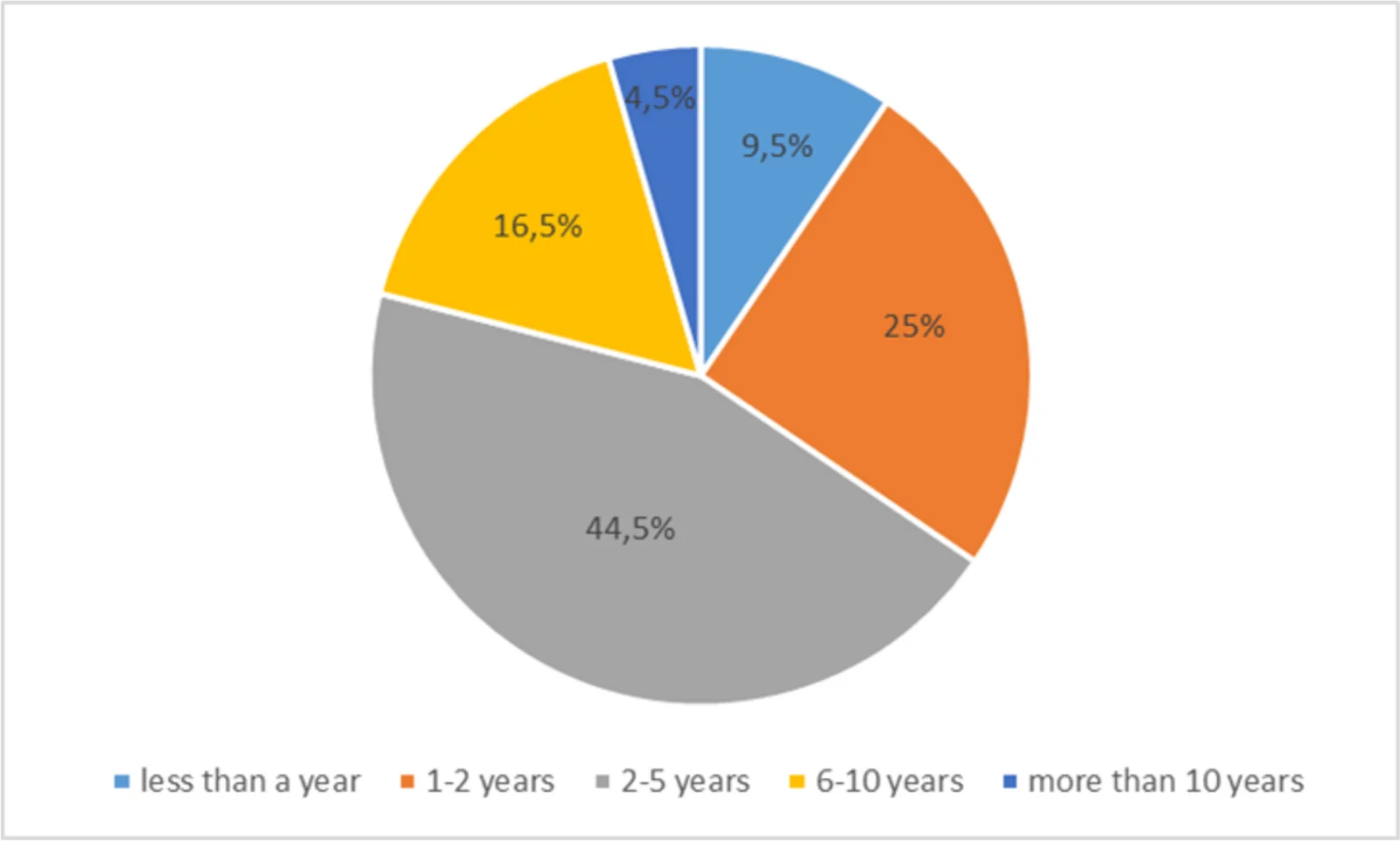
The percentage of the surgeons’ experience with the use of ICG in the minimally invasive elective colorectal resections classified per years of experience

**Fig. 5:**
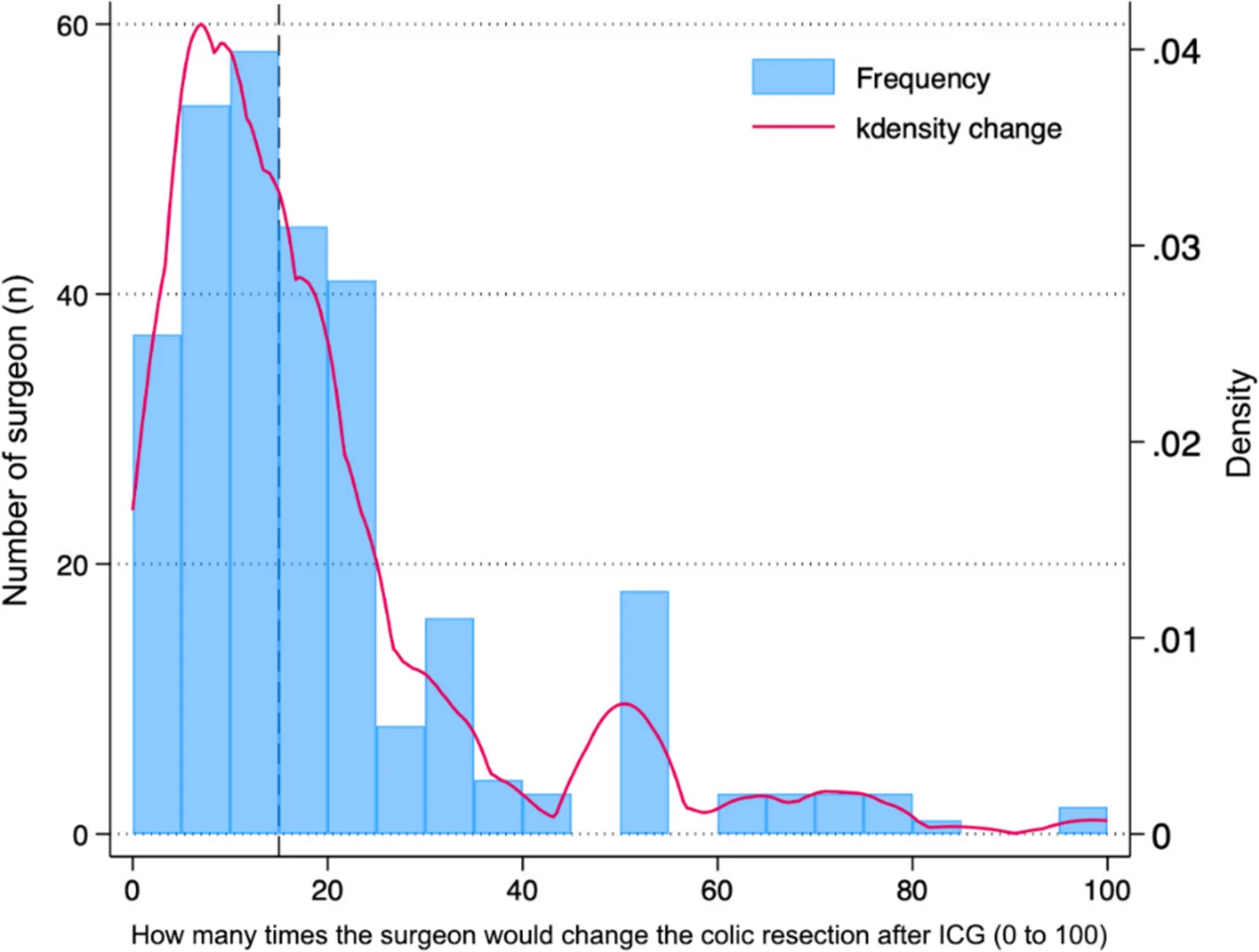
Distribution of surgical decision-making changes following ICG fluorescence. The histogram and kernel density plot illustrate the frequency with which surgeons would change their colic resection plane after ICG assessment. The total of the surgeons who replied to this question was 287

The survey shows a widespread availability of ICG in various hospitals: 949 (76%) of the surgeons confirmed availability of ICG in their hospital. Of these, 833 (67%) use ICG for the assessment of anastomotic integrity in colorectal surgery. 606 (50% overall) surgeons have ICG available in the hospital most of the time and 475 (39%) always, 120 (10%) depending on what type of colorectal resection and only 12 (1%) surgeons replied that ICG is never available. The survey showed a wide heterogeneity in the type of the system and the most used is the Karl Storz system 423 (34.5%) (Table 2). Most of the respondents 567 (69.5%) had an established experience with ICG between 1 and 5 years and only 78 (9.5%) say less than 1 year, meanwhile 173 (21%) use ICG more than 5 years (Fig. 4). The use of ICG is a familiar practice to surgeons who feel quite confident in its use: 670 of them (54%) feel moderately confident, 437 (35%) strongly confident and only 138 (11%) not very confident. On the other hand, there is concordance for the time and the dose to use: 72% of respondents inject ICG at 1 min or just before and 961 (72%) use standard dose with different concentration (Table 2). Only 287 (21%) respondents specified the rate of changing the resection line using ICG (Fig. 5). Most of them change the line of transection between 4 and 50 times. The data show, limitless to the sample, that ICG is a tool that surgeons rely on before resection of the bowel.

**Table 2:** The use of indocyanine green (ICG)

		Num	%
Availability of ICG			
Is ICG available at your institution?	Yes	949	76
	No	304	24
Do you use ICG in colorectal surgery?	Yes	833	67
	No	455	33
What type of fluorescence system do you have available?	Karl Storz	423	34.5
	Olympus	287	23.4
	Novadaq	31	2.5
	Stryker	209	17
	Intuitive/Firefy	211	17
	Aesculap	11	0.9
	Other	54	4.4
If you have the fluorescence system available at your institution, is it available for use?	Always	475	39
	Most of the times	606	50
	Depending of the site of the column	120	10
	Never		
		12	v
Confidence with ICG system			
How extensive is your experience with the use of ICG in the minimally invasive elective colorectal resections?	Less than a year	78	9.5
	1–2 years	203	24.9
	2–5 years	364	44.5
	6–10 years	136	16.5
	More than 10 years	37	4.5
How confident do you feel in using the ICG in colorectal resection?	Strongly confident	437	35
	Moderately confident	670	54
	Not very confident	138	11
Operating room protocol			
At what time point do you use ICG?	Before dividing the bowel	337	40
	Immediately after division the of the bowel	58	7
	After the division of the bowel—just before creating the anastomosis	208	25
	After creation of anastomosis	143	17
	Two times—before the anastomosis and after the creation of anastomosis	91	11
	Other	5	0.6
ICG dosing	Standard single dose	589	71
	Depending on the weight of the patient	245	29
Standard single dose	5 mg	297	30
	10 mg	362	36.5
	15 mg	159	16
	20 mg	64	6.5
	25 mg	110	11
Depending on the weight of the patient	0.1 mg/kg	63	14.5
	0.2–0.3 mg/kg	318	73
	> 0.3 mg/kg	54	12.5
What type of mode do you use?	Black/white light	310	28
	SPY fluoresce way	233	21
	Overlay fluorescence mode	390	36
	Color-segmented fluorescence mode	163	15
How do you evaluate the adequacy of the perfusion by using ICG?	Subjective evaluation	780	93
	The use of software	49	6
	Artificial intelligence	7	0.8
	Other	3	0.3
The method of injection	Iv bolus	797	95
	Through infusion set	42	5
Opinion about ICG			
Do you think that the use of ICG reduces the risk of anastomotic leak?	Yes	759	88
	No	100	12
Do you think that ICG use will become a standard procedure in elective colorectal surgery?	Yes	786	91
	No	72	9
Do you think that the use of ICG reduces the percentage of protective ileostomy in rectal surgery?	Yes	384	45
	No	474	55
Do you think that the use of ICG reduces the percentage of leak in EMERGENCY colorectal surgery?	Yes	567	66
	No	293	34

### Perceived impact of ICG on clinical practice

A total of 786 respondents (91%) reported that intraoperative colorectal perfusion assessment is expected to become standard in elective colorectal surgery. Additionally, 567 respondents (66%) anticipated its use in emergency procedures. However, the majority (474; 55%) believed that ICG does not reduce the rate of protective ileostomy in rectal surgery even if 759 surgeons (88%) indicated that ICG fluorescence may help reduce the risk of anastomotic leakage (Table 2).

### Flexible endoscopy for intraoperative assessment of anastomotic integrity

As highlighted above, the routine use of intraoperative endoscopy by surgeons plays an important role in assessing anastomotic integrity. In the corresponding section of the survey, 1216 out of 1367 participants (89%) responded to questions regarding their use of flexible endoscopy. Half of the respondents 619 (51%) reported routinely using flexible endoscopy in their clinical or surgical practice; however, only 409 (35%) had received formal endoscopic training (Table 3). Among those who had been trained, 51.6% indicated that endoscopy was a mandatory component of their surgical training, while 29.6% had participated in a dedicated training program. The question regarding the availability of endoscopic equipment in hospitals was answered by 1,200 respondents. Among them, 395 (32.9%) reported having access to flexible endoscopy, and 125 (10.4%) reported having only rigid endoscopy available. Furthermore, 433 respondents (36%) indicated that both instruments were available in their hospitals, while 267 (22.2%) reported having neither type of endoscopic equipment.

**Table 3:** The use of flexible endoscopy (FE)

		Num	%
General questions			
Do you use flexible endoscopy in your clinical and surgical activity?	Yes	619	51
	No	597	49
How long have you been using flexible endoscopy (years)?	0	15	25
	1–5	22	27
	6–10	8	13
	>10	15	15
How many endoscopic procedures did you perform in general?	0	17	29
	1–10	5	8.5
	11–50	6	10
	51–100	9	15.2
	>100	22	37.3
Is endoscopy available for surgeons at your institution?	Flexible endoscopy	395	32.5
	Rigid endoscopy	125	10
	Both	433	35.5
	Not available	267	22
What type of flexible endoscopy system do you have available?	Karl Storz	259	22
	Olympus	746	62
	Pentax	136	11
	Other	71	6
Do you routinely use endoscopy to check the anastomosis in the operating room?	Yes	279	23
	No	938	77
Do any other members of your surgical team perform endoscopy if you do not?	Yes	298	32
	No	634	68
If you do not use flexible endoscopy, what are the reasons?	No access	223	17
	Not properly trained	246	19
	It is only performed by the gastroenterologists	373	28
	I use other type of technique to check the anastomosis	258	20
	Not able to perform endoscopy due to hospital policy	62	5
	I don’t think it’s useful	125	9
	Other	24	2
If you have flexible endoscopy available at your institution, when is it available to use?	Always	331	29
	Most of the time intra-operatively	280	24
	Sometimes	218	19
	Rarely	193	17
	Never	123	11
Endoscopy in operating room			
How many FE procedures did you perform in the OR?	0	38	14
	1–10	61	22
	11–50	78	28
	51–100	49	18
	>100	49	18
Beside intraoperative colonoscopy, Do you also use endoscopy for other indications? (multiple choice)	Diagnostic EGD	470	15
	Intraoperative EGD	408	13
	Treatment of surgical complications	508	16.5
	Interventional procedure		
	ERCP	397	13
	Diagnostic colonoscopy	304	10
	Therapeutic colonoscopy	615	20
	No	503	16
	Other	321	10
		23	0.8
Did you receive any training prior to performing FE in the OR?	Yes	409	35
	No	770	65
Do your usually use operative endoscopy to resolve anastomotic complications?	Never	294	25
	Sometimes	472	39
	Always	37	3
	Depends on the type of complication	392	33
What type of treatment do you use in case of dehiscence of the anastomosis during intraoperative endoscopic assessment?	Endoscopic clips	357	16
	Over-the-Scope Clip (OTSC)	240	11
	Internal drainage (pig tails)	222	10
	Stents	245	11
	Endoscopic suturing	143	6
	Endoscopic vacuum systems	370	16
	Treatment depends on individual circumstances	680	30
Opinion about flexible endoscopy			
Do you think that FE use will become a standard procedure in elective colorectal surgery?	Yes	567	47
	No	635	53
Do you think that the use of FE reduces the percentage of protective ileostomy in rectal surgery?	Yes	377	31
	No	829	69
Do you think that the use of FE reduces the risk of anastomotic leak?	Yes	573	48
	No	635	52
Do you think that the use of FE reduces the percentage of leak in colorectal surgery in EMERGENCY surgery?	Yes	385	32
	No	817	68

Most surgeons 938 (77%) did not assess the anastomosis with flexible endoscopy in the operating room for various reasons. The majority 373 (28.5%) reported that flexible endoscopy is performed exclusively by gastroenterologists in their hospitals. Other common reasons for not performing flexible endoscopy included the use of alternative methods to assess anastomotic integrity 258 (19.7%) and a lack of adequate training 246 (19%). 223 respondents (17%) indicated that they do not perform flexible endoscopy due to limited access to the necessary equipment (Table 3).

**Fig. 6:**
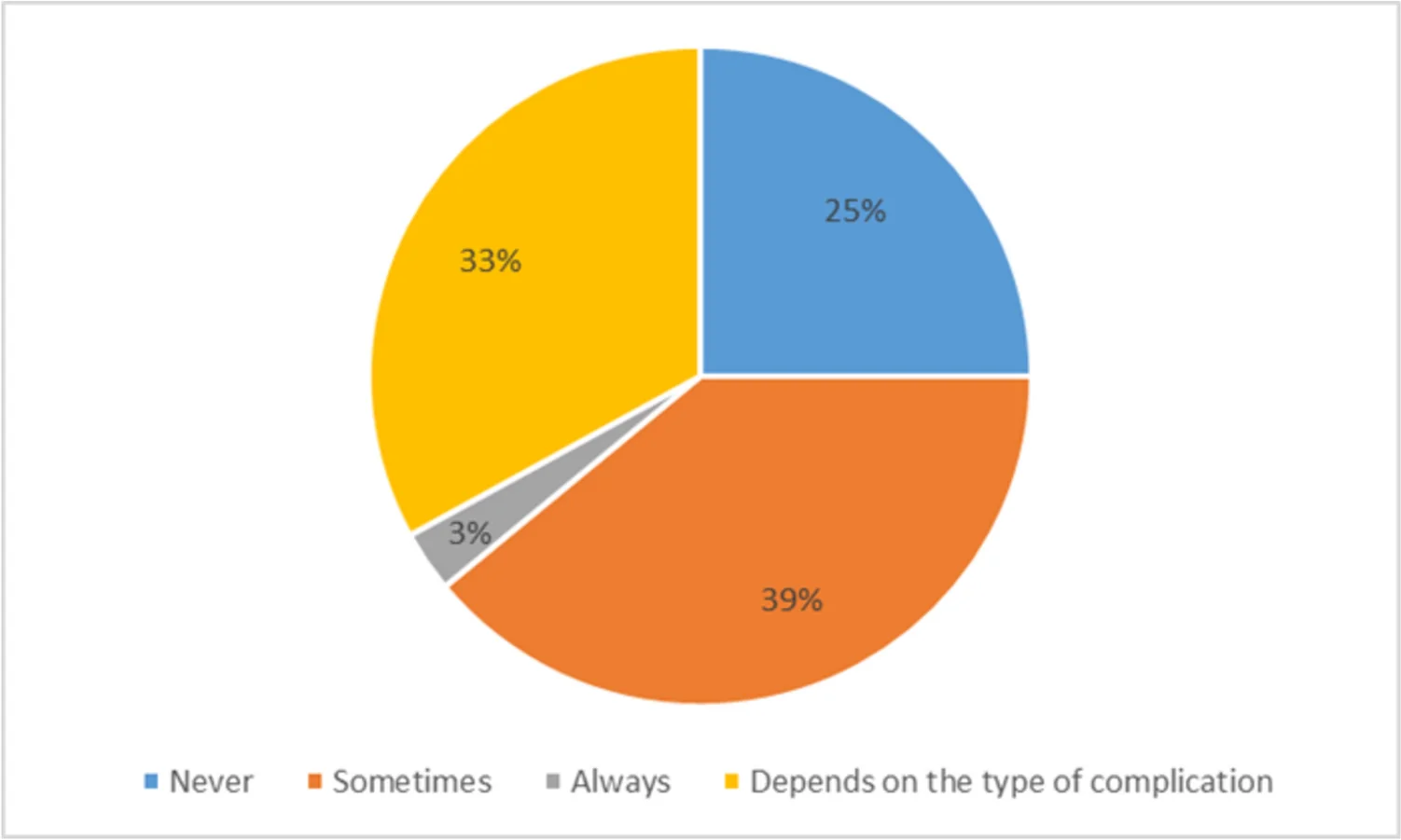
The frequency with which the surgeons use operative endoscopy to resolve anastomotic complication

**Fig. 7:**
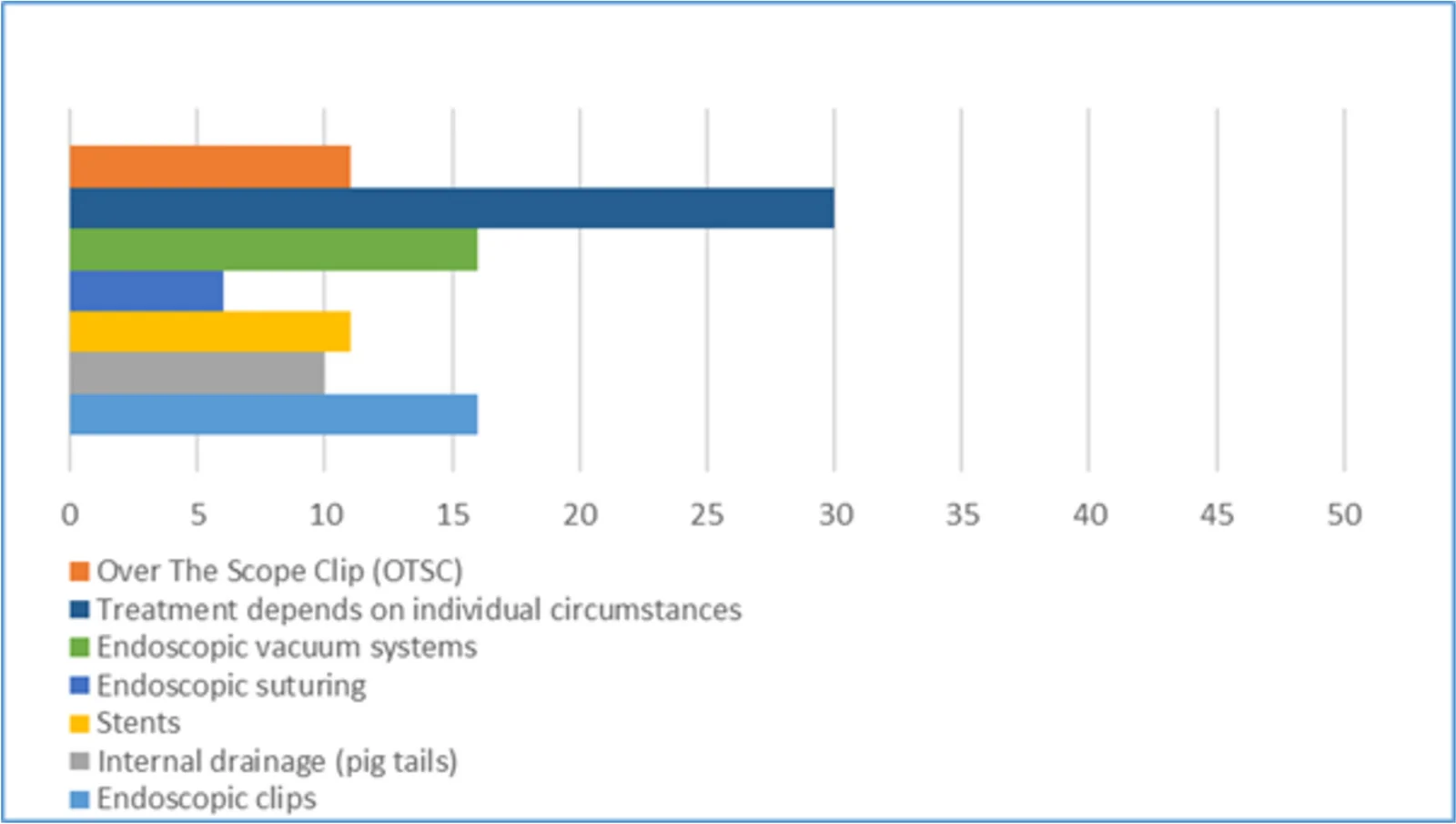
The percentage of the different types of endoscopic treatment used in the operative room by the surgeons in case of colorectal anastomotic leak

Anastomotic complications remain among the most feared, highlighting the importance of effective management. Among 1,195 respondents, 294 (24.6%) reported never using flexible endoscopy (FE) for these complications, 472 (39.5%) used it occasionally, 37 (3.1%) used it consistently, and 392 (32.8%) indicated that their use of FE depends on the type of complication (Fig. 6). Among those who do use FE, considerable heterogeneity was observed in the choice of endoscopic treatment: for most surgeons 680 (30%), the approach depends on the circumstances, followed by endoscopic clips and vacuum-assisted systems 370 (16%), stents 245 (11%), over-the-scope clips (OTSC) 240 (11%), internal drainage with pigtails 222 (10%), and endoscopic suturing 143 (6%) (Fig. 7).

### Perceived impact of FE on clinical practice

A total of 567 (47%) respondents replied that the use of FE will become a standard procedure in elective colorectal surgery. Similar, 573 (48%) of the respondents think that it will reduce the risk of anastomotic leak. For emergency surgery, only 385 (32%) of the respondents think this risk will be reduced. For rectal surgery, 377 (31%) of the respondents indicated that FE reduces the percentage of protective ileostomy (Table 3).

## Discussion

This global survey provides a contemporary overview of surgical practices and anastomotic safety measures in colorectal surgery. Although ICG is widely available and more frequently adopted, both techniques lack standardized protocols, and flexible endoscopy remains limited by training and access. The heterogeneous adoption of perfusion assessment techniques, ranging from conventional visual inspection to ICG fluorescence and intraoperative endoscopy, reflects both evolving technology and variation in local protocols or resource availability.

While most surgeons report acceptable anastomotic leak rates following colonic surgery, outcomes in rectal surgery remain more variable, underscoring the clinical relevance of reliable intraoperative perfusion assessment methods. This study shows the current use of ICG in different parts of the world, indicating a widespread availability of ICG technology for 76% of the respondents. Anastomotic leak after colorectal resection increases morbidity and mortality, with risks like permanent stoma, local recurrence, reduced cancer survival, and poor function and quality of life. Reducing leak rates is therefore a key goal. In the literature different techniques are reported such as leak test, endoscopy, perfusion checks, and fluorescence imaging.

ICG fluorescence imaging is a relatively recent technology that has progressively become part of standard clinical practice over the past decade, supported by increasing clinical trial evidence [17–19]. Most surgeons 364 (44.5%) reported 2–5 years of experience with ICG and expressed confidence in its use (Fig. 4) [20]. However, lack of standardization and interuser variability remain important issues, particularly regarding timing and dosage of injection, as also reported in the literature [21, 22].

The majority of respondents 603 (72%) agreed on performing ICG assessment at the time of resection to evaluate perfusion of both intestinal ends before performing the anastomosis. Nevertheless, no consensus emerged on the optimal dose for perfusion assessment: 589 (71%) reported using a fixed standard dose, whereas 245 (29%) adjusted the dose according to patient weight. Furthermore, perfusion assessment remains largely subjective. Ongoing research is exploring artificial intelligence-based tools and software solutions to reduce interobserver variability and enable more objective interpretation of ICG angiography in future [23, 25, 26].

Finally, surgeons’ perceptions regarding the usefulness of ICG in colorectal surgery were largely positive. A total of 786 respondents (91%) believed that ICG will become standard practice in colorectal surgery, and 759 (88%) considered it effective in reducing the risk of anastomotic leakage. Overall, 780 surgeons (93%) expressed views consistent with the available literature. Several studies have shown that ICG use in colorectal surgery is associated with a reduced risk of anastomotic leakage related to impaired bowel perfusion. However, further randomized controlled trials are needed to provide robust evidence of its clinical efficacy [27–30]. However, heterogeneity in study design, patient selection, and intraoperative ICG protocols continues to limit interpretation and comparison across studies.

This study highlights some critical disparities in the use, availability, and training regarding FE among surgeons. Despite consensus on its clinical value, only 619 (51%) of respondents reported routine use, indicating a significant gap between recognition and practice. Equipment availability remains a major obstacle, with 267 (22.2%) of participants not having access to any endoscopic tools. Routine intraoperative evaluation of anastomoses by FE was only performed by 279 (23%), suggesting underutilization despite growing evidence that FE may improve intraoperative detection of anastomotic defects and reduce postoperative complications [10, 13].

A key barrier to wider adoption appears to be the training. Only 409 (35%) of respondents reported having received adequate training, underscoring the important need for structured educational programs. Interestingly, this finding contrasts with other surgical specialties such as urology and thoracic surgery, where surgeons continue to routinely perform and maintain endoscopic skills as part of standard clinical practice. Previous surveys among surgeons from different healthcare systems have consistently demonstrated that, although endoscopy is considered an essential component of colorectal and general surgical practice, procedural exposure during residency remains limited and highly variable [31, 32]. The progressive separation between gastrointestinal surgery and endoscopy may be multifactorial and has previously been attributed to increasing subspecialization, institutional organization in which gastroenterologists perform the majority of endoscopic procedures, limited access to dedicated training opportunities, time constraints during residency, and competition for procedural exposure [31–33]. While diagnostic and therapeutic colonoscopy were the most common indications for FE, a considerable portion of respondents 294 (25%) never used FE to treat anastomotic complications, conditions in which early endoscopic intervention could be beneficial. Many indicated the need for formal education, with over half citing mandatory training during surgical education. Given the expanding role of minimally invasive and endoluminal techniques in colorectal surgery, restoring surgical ownership and competency in flexible endoscopy may become essential for colorectal practice.

These findings emphasize the need to integrate FE more comprehensively into surgical curricula and institutional practice. Beyond technical assessment alone, surgeon-performed FE may improve continuity of care by allowing colorectal surgeons to independently evaluate anastomotic integrity, guide intraoperative decision-making, and manage postoperative complications without reliance on additional specialists [32, 33]. Recent efforts have focused on developing structured perioperative FE assessment protocols and grading systems for anastomotic ischemia and integrity, which may improve reproducibility and facilitate broader implementation in colorectal surgery [15]. Standardizing access, reinforcing multidisciplinary collaboration, and establishing robust training pathways are essential steps toward optimizing surgical care and improving patient safety.

The intraoperative check of anastomosis with the aim to reduce AL is widely adopted in colorectal surgery. However, both FE and ICG currently lack standardized protocols regarding indications, timing, interpretation, and integration into intraoperative decision-making. This absence of standardization likely contributes to the heterogeneity observed across studies and may partly explain why reductions in AL rates reported in randomized trials are less pronounced in routine clinical practice [14]. Standardization of these methods is imperative for routine use [14, 34]. Toward that end, the quadruple anastomotic assessment was introduced to include donut inspection, air leak testing, FE, and ICG perfusion evaluation [16]. Such combined approaches may be particularly valuable because perfusion assessment alone cannot identify all technical or intraluminal defects, whereas FE provides direct visualization of the anastomosis itself [10, 16]. This technique has subsequently been validated by other surgeons [35].

Unfortunately, as this study discovered, one potential barrier to adoption of the quadruple assessment technique is lack of education and training and/or equipment availability for intraoperative FE. This problem could potentially be overcome by EAES, SAGES, and other organizations offering education. However, even if education in FE were universal, resource availability may remain a challenge. Another problem is lack of consensus on ICG dosing. This issue has been addressed by the International Society of Fluorescence-Guided Surgery (ISFGS) by publishing a Delphi-driven dose chart on the ISFGS website [34, 36]. In addition, the marked heterogeneity in ICGA timing, dosing, interpretation, and integration into surgical decision-making observed in this survey likely represents an important limitation to demonstrating its full clinical benefit in daily practice. Although randomized controlled trials have reported relative reductions in anastomotic leakage rates ranging between 10 and 20%, real-world data suggest that these effects may be substantially smaller in routine clinical practice, potentially due to inconsistent application and lack of standardized protocols [14, 24].

This study has several limitations. First, response bias may have influenced the results, as not all respondents completed every question, particularly those related to the FE section. Consequently, some questions were excluded from analysis because the number of responses was insufficient to allow meaningful conclusions. Second, not all participants were colorectal surgeons, although the survey focused on the use of these technologies in colorectal surgery. Finally, because the survey was disseminated beyond scientific societies such as EAES and SAGES, it was not possible to determine the total number of surgeons invited to participate, precluding calculation of an overall response rate.

## Conclusion

This international survey highlights substantial variability in the use of ICG fluorescence and flexible endoscopy for assessing colorectal anastomotic integrity. Although ICG is widely available and more frequently adopted, both techniques lack standardized protocols, and flexible endoscopy remains limited by training and access. These findings underscore the need for structured education and prospective studies to define evidence-based workflows that can enhance anastomotic safety in colorectal surgery.

## Supplementary Information

Below is the link to the electronic supplementary material.

Supplementary file1 (PDF 56 kb)
