# Correction: Colorectal anastomotic safety assessment using ICG fluorescence and flexible endoscopy (COLOSSEUM): a global survey of 1367 surgeons

**DOI:** 10.1007/s00464-026-13282-7

**Published:** 2026-08-10

**Authors:** A. Belvedere, E. Licardie, D. Sochorova, L. Boni, M. Chand, S. Perretta, S. Wexner, T. H. A. Arulampalam, S. Morales-Conde, S. F. Hardon

**Affiliations:** 1 Department of General and Emergency Surgery, IRCCS Azienda Ospedaliero-Universitaria Di Bologna, Bologna, Italy; 2 Department of General and Digestive Surgery, University Hospital Virgen Macarena, University of Sevilla, Seville, Spain; 3 Department of Surgery, Tomas Bata Hospital, Zlin, Czech Republic; 4 Department of HPB and Transplant Surgery, Freeman Hospital, Newcastle Upon Tyne, UK; 5 Department of General & Minimally Invasive Surgery, Fondazione IRCCS – Ca’ Granda - Ospedale Maggiore Policlinico di Milan, Milan, Italy; 6 Department of Advanced Minimally-Invasive Surgery, University College London Hospital, London, UK; 7 Department of Surgery, University Hospital, Strasbourg, France; 8 Department of Colorectal Surgery, Ellen Leifer Shulman and Steven Shulman Digestive Disease Center, Cleveland Clinic Florida, Weston, FL USA; 9 Department of Colorectal Surgery, Medstar Georgetown University Health, Washington, DC USA; 10 School of Medicine, Anglia Ruskin University, Chelmsford, UK; 11 Department of Surgery, Amsterdam UMC, De Boelelaan 1117, 1081HV Amsterdam, The Netherlands

**Correction to: Surgical Endoscopy** **https://doi.org/10.1007/s00464-026-12974-4**

The original online version of this article was revised to correct the presentation of the name of coauthor S. Morales-Conde.

The original article has been corrected.

