# Supplementary material for: Colorectal anastomotic safety assessment using ICG fluorescence and flexible endoscopy (COLOSSEUM): a global survey of 1367 surgeons
Source: Surg Endosc. 2026 Jul 13;40(9):7885–901. doi: 10.1007/s00464-026-12974-4 (PMC13582295; doi:10.1007/s00464-026-12974-4)
Supplement: Supplementary file 1 [file 464_2026_12974_MOESM1_ESM.pdf]

Colosseum Survey

Dear colleagues,We kindly ask a few minutes or your time.The COLOrectal Surgical Safety Enhanced Using Mixed Methods (COLOSSEUM) project was launched as part of the EAES Rising Stars Academy 2023 in Rome.This study aims to evaluate anastomosis assessment by perfusion (ICG) and integrity (flexible endoscopy).The survey takes 2 minutes and all respondents are invited as a collaborator in this and future projects of our study group.Kind regards,Sem Hardon, Angela Belvedere, Dana Sochorova, Eugenio LicardieOn behalf of COLOSSEUM study group(T. Arulampalam, L. Boni, M. Chand, S. Morales-Conde, S. Perretta, and S. Wexner)

General questions

1 Age

____

2 Country

____

3 Type of hospital

- ◯ Private hospital
- ◯ Public hospital
- ◯ University hospital

4 Have you finished general surgery training?

- ◯ Yes
- ◯ No

5 Have you also trained in colorectal surgery fellowship?

- ◯ Yes
- ◯ No

6 Years since finishing training

____

7 Main specialty:

- ☐ General Surgeon
- ☐ Colorectal surgeon
- ☐ Upper GI
- ☐ HPB
- ☐ Bariatric surgeon
- ☐ Abdominal wall surgeon
- ☐ Other

8 How many elective MINIMALLY invasive colorectal resections (both for benign and oncological indications) are performed at your institution per year in TOTAL?

- ☐ 1-20
- ☐ 21-50
- ☐ 51-75
- ☐ 76-100
- ☐ more than 100

9 How many elective OPEN colorectal resections (both for benign and oncological indications) are performed at your institution per year in TOTAL?

- ☐ 1-20
- ☐ 21-50
- ☐ 51-75
- ☐ 76-100
- ☐ more than 100

10 What percentage of your elective colorectal resections (both for benign and oncological indications) are done as open cases?

- ◯ less than 10%
- ◯ 11% -25%
- ◯ 26% -50%
- ◯ 51% -75%
- ◯ 76% -89%
- ◯ more than 90%

11	What percentage of your elective colorectal resections (both for benign and oncological indications) are done as laparoscopic cases?	◯ less than 10%<br>◯ 11% -25%<br>◯ 26% -50%<br>◯ 51% -75%<br>◯ 76% -89%<br>◯ more than 90%
12	What percentage of your elective colorectal resections (both for benign and oncological indications) are done as robotic cases?	◯ none<br>◯ less than 10%<br>◯ 11% -25%<br>◯ 26% -50%<br>◯ 51% -75%<br>◯ 76% -89%<br>◯ more than 90%
13	How many anastomotic leaks after elective RIGHT colon resection occur per year in your surgical unit?	◯ less than 5%<br>◯ 5% -10%<br>◯ 11% -15%<br>◯ 16% -20%<br>◯ more than 20%
14	How many anastomotic leaks after elective LEFT colon resection occur per year in your surgical unit?	◯ less than 5%<br>◯ 5% -10%<br>◯ 11% -15%<br>◯ 16% -20%<br>◯ more than 20%
15	How many anastomotic leaks after elective RECTAL surgery occur per year in your surgical unit?	◯ less than 5%<br>◯ 5% -10%<br>◯ 11% -15%<br>◯ 16% -20%<br>◯ more than 20%
16	How do you currently check the perfusion of the colon BEFORE the anastomosis?	☐ Visual inspection of the colon "color"<br>☐ ICG<br>☐ Hyperspectral imaging<br>☐ Visually checking the pulse of the artery branch in the mesentery<br>☐ Palpatory checking the pulse of the artery branch in the the mesentery (in open cases)<br>☐ Check if the colon blind ends bleeds after making a small cut<br>☐ Endoscopic inspection<br>☐ other
17	If other (note)	__________________________________
18	How do you currently check the perfusion of the colon AFTER the anastomosis?	☐ visual inspection of the colon "color"<br>☐ ICG<br>☐ hyperspectral imaging<br>☐ intraoperative endoscopy<br>☐ don't check anymore<br>☐ other
19	If other (note)	__________________________________

ICG questions

20 Is ICG available at your institution?
- ◯ Yes
- ◯ No

21 Do you use ICG in colorectal surgery?
- ◯ Yes
- ◯ No

22 What type of fluorescence system do you have available?
- ☐ Karl Storz
- ☐ Olympus
- ☐ Novadaq
- ☐ Stryker
- ☐ Intuitive/Firefy
- ☐ Aesculap
- ☐ Other

23 If other (note)

____

24 If you have the fluorescence system available at your institution, is it available for use:
- ◯ always
- ◯ most of the time
- ◯ depending of the site of colon-rectum
- ◯ never

25 If " depending of the site of colon-rectum", specify:
- ◯ Right colon
- ◯ Left colon
- ◯ Partial colon resection (splenic flexure)
- ◯ Rectum

26 How extensive is your experience with the use of ICG in the minimally invasive elective colorectal resections?
- ◯ less than a year
- ◯ 1-2 years
- ◯ 2-5 years
- ◯ 6-10 years
- ◯ more than 10 years

27 Have you received any formal training regarding the use of ICG in colorectal surgery?
- ☐ EAES course
- ☐ European Surgical Society of Oncology course
- ☐ during my fellowship
- ☐ at my institution (by my supervisor)
- ☐ by industry
- ☐ ISFGS course
- ☐ other
- ☐ no

28 If other, (note)

29 How confident do you feel in using the ICG in colorectal resection?
- ◯ strongly confident
- ◯ moderately confident
- ◯ not very confident

30 If you use ICG, At what time point do you use ICG?
- ☐ before dividing the bowel
- ☐ immediately after division the of the bowel
- ☐ after the division of the bowel - just before creating the anastomosis
- ☐ after creation of anastomosis
- ☐ Two times - before the anastomosis and after the creation of anastomosis
- ☐ other

31	If other (note)	__________________________________
32	ICG dosing	☐ Standard single dose<br>☐ Depending on the weight of the patient
33	Standard single dose	◯ 5 mg<br>◯ 10 mg<br>◯ 15 mg<br>◯ 20 mg<br>◯ 25 mg
34	Depending on the weight of the patient	◯ 0.1 mg/kg<br>◯ 0.2-0.3 mg/kg<br>◯ >0.3 mg/kg
35	What type of mode do you use?	☐ black/white light<br>☐ SPY fluoresce way<br>☐ Overlay Fluorescence Mode<br>☐ Color-Segmented Fluorescence Mode
36	How do you evaluate the adequacy of the perfusion by using ICG?	☐ subjective evaluation<br>☐ the use of software<br>☐ artificial intelligence<br>☐ other
37	If other (note)	__________________________________
38	The method of injection	◯ iv bolus<br>◯ throught infusion set (longer administration)
39	At what time point do you evaluate the fluorescence ?	◯ When reaching the peak before the first minute<br>◯ At 1 minutes<br>◯ At 3 minutes
40	Do you know the rate of changing the transection site using ICG ?	◯ Yes I know<br>◯ I don´t know
41	Indicate the percentage	0 50 100<br>*(Place a mark on the scale above)*
42	Do you think that the use of ICG reduces the risk of anastomotic leak?	◯ Yes<br>◯ No
43	Do you think that ICG use will become a standard procedure in elective colorectal surgery?	◯ Yes<br>◯ No
44	Do you think that the use of ICG reduces the percentage of protective ileostomy in rectal surgery?	◯ Yes<br>◯ No
45	Do you think that the use of ICG reduces the percentage of leak in EMERGENCY colorectal surgery	◯ Yes<br>◯ No

Endoscopy survey

46	Do you use flexible endoscopy in your clinical and surgical activity?	◯ Yes<br>◯ No
47	How long have you been using flexible endoscopy (years)?	◯ 0<br>◯ 1-5<br>◯ 6-10<br>◯ >10
48	How many endoscopic procedures did you perform in general?	◯ 0<br>◯ 1-10<br>◯ 11-50<br>◯ 51-100<br>◯ >100
49	Is endoscopy available for surgeons at your institution?	◯ Flexible endoscopy<br>◯ Rigid endoscopy<br>◯ Both<br>◯ Not available
50	What type of flexible endoscopy system do you have available?	☐ Karl Storz<br>☐ Olympus<br>☐ Pentax<br>☐ Other
51	If other (note)	__________________________________
52	Do you routinely use endoscopy to check the anastomosis in the operating room?	◯ Yes<br>◯ No
53	Do any other members of your surgical team perform endoscopy if you do not?	◯ Yes ◯ No
54	If you do not use flexible endoscopy, what are the reasons?	☐ No access<br>☐ Not properly trained<br>☐ It is only performed by the gastro-enterologists<br>☐ I use other type of technique to check the anastomosis<br>☐ Not able to perform endoscopy due to hospital policy<br>☐ I don't think it's useful<br>☐ Other
55	If other (note)	__________________________________
56	If you have flexible endoscopy available at your institution, when is it avaible to use intra-operatively?	◯ Always<br>◯ Most of the time<br>◯ Sometimes<br>◯ Rarely<br>◯ Never

57 For which parts of the colon and the rectum do you perform endoscopy intra-operatively?

- ◯ Right colon
- ◯ Left colon
- ◯ Rectum
- ◯ Left colon and rectum
- ◯ All of the above

58 Do you use flexible endoscopy to check the anastomotic integrity during minimally invasive elective colorectal resections?

- ☐ Always
- ☐ Most of the time
- ☐ Sometimes
- ☐ Only if there is a suspicion of anastomotic defect
- ☐ Only if there is a suspicion of anastomotic bleeding
- ☐ It depends on the part of the colon and rectum

59 How many FE procedures did you perform in the OR?

- ◯ 0
- ◯ 1-10
- ◯ 11-50
- ◯ 51-100
- ◯ >100

60 Beside intraoperative colonoscopy, Do you also use endoscopy for other indications?

- ☐ Diagnostic EGD
- ☐ Intraoperative EGD
- ☐ Treatment of surgical complications
- ☐ Interventional procedure
- ☐ ERCP
- ☐ Diagnostic colonoscopy
- ☐ Therapeutic colonoscopy
- ☐ No
- ☐ Other

61 If other, note

____

62 Did you receive any training prior to performing FE in the OR?

- ◯ Yes
- ◯ No

63 If yes, in what way?

- ◯ mandatory part of my surgical training
- ◯ EAES course
- ◯ part of my official full endoscopy training
- ◯ other

64 If other (note)

____

65 Do you usually use operative endoscopy to resolve anastomotic complications?

- ◯ never
- ◯ sometimes
- ◯ always
- ◯ depends on the type of complication

66 What type of treatment do you use in case of dehiscence of the anastomosis during intraoperative endoscopic assessment?

- ☐ Endcoscopic clips
- ☐ Over The Scope Clip (OTSC)
- ☐ Internal drainage (pig tails)
- ☐ Stents
- ☐ Endoscopic suturing
- ☐ Endoscopic vacuum systems
- ☐ Treatment depends on individual circumstances

67	Do you think that the use of flexible endoscopy reduces the risk of anastomotic leak?	◯ Yes<br>◯ No
68	Do you think that FE use will become a standard procedure in elective colorectal surgery?	◯ Yes<br>◯ No
69	Do you think that the use of FE reduces the percentage of protective ileostomy in rectal surgery?	◯ Yes<br>◯ No
70	Do you think that the use of FE reduces the percentage of leak in colorectal surgery in EMERGENCY surgery?	◯ Yes<br>◯ No
71	Do you like to be involved as a collaborator in this project and potential future projects?	◯ Yes<br>◯ No
72	If yes, please enter your name:	______________________________<br>((initials and surname as J.A. Smith))
73	If yes, please enter your email:	______________________________
